# Identification of new EphA4 inhibitors by virtual screening of FDA-approved drugs

**DOI:** 10.1038/s41598-018-25790-1

**Published:** 2018-05-09

**Authors:** Shuo Gu, Wing-Yu Fu, Amy K. Y. Fu, Estella Pui Sze Tong, Fanny C. F. Ip, Xuhui Huang, Nancy Y. Ip

**Affiliations:** 1 Division of Life Science, The Hong Kong University of Science and Technology, Clear Water Bay, Hong Kong, China; 2 Molecular Neuroscience Center, The Hong Kong University of Science and Technology, Clear Water Bay, Hong Kong, China; 3 State Key Laboratory of Molecular Neuroscience, The Hong Kong University of Science and Technology, Clear Water Bay, Hong Kong, China; 4 Department of Chemistry, The Hong Kong University of Science and Technology, Clear Water Bay, Hong Kong, China; 5 Guangdong Provincial Key Laboratory of Brain Science, Disease and Drug Development, HKUST Shenzhen Research Institute, Shenzhen, China

## Abstract

The receptor tyrosine kinase, erythropoietin-producing hepatocellular A4 (EphA4), was recently identified as a molecular target for Alzheimer’s disease (AD). We found that blockade of the interaction of the receptor and its ligands, ephrins, alleviates the disease phenotype in an AD transgenic mouse model, suggesting that targeting EphA4 is a potential approach for developing AD interventions. In this study, we identified five FDA-approved drugs—ergoloid, cyproheptadine, nilotinib, abiraterone, and retapamulin—as potential inhibitors of EphA4 by using an integrated approach combining virtual screening with biochemical and cellular assays. We initially screened a database of FDA-approved drugs using molecular docking against the ligand-binding domain of EphA4. Then, we selected 22 candidate drugs and examined their inhibitory activity towards EphA4. Among them, five drugs inhibited EphA4 clustering induced by ephrin-A in cultured primary neurons. Specifically, nilotinib, a kinase inhibitor, inhibited the binding of EphA4 and ephrin-A at micromolar scale in a dosage-dependent manner. Furthermore, nilotinib inhibited the activation of EphA4 and EphA4-dependent growth cone collapse in cultured hippocampal neurons, demonstrating that the drug exhibits EphA4 inhibitory activity in cellular context. As demonstrated in our combined computational and experimental approaches, repurposing of FDA-approved drugs to inhibit EphA4 may provide an alternative fast-track approach for identifying and developing new treatments for AD.

## Introduction

Erythropoietin-producing hepatocellular (Eph) receptors, the largest family of receptor tyrosine kinases, are involved in a diverse spectrum of cellular processes^1^. Eph receptors are activated by binding with their transmembrane ligands, ephrins, to generate bidirectional signals via cell–cell interactions^1,2^. The Eph receptors are subdivided into EphAs (EphA1–EphA8 and EphA10) and EphBs (EphB1–EphB4 and EphB6). EphA receptors preferentially bind to their cognate ligands, ephrin-As (ephrin-A1–ephrin-A5), which are anchored to the membrane via glycosylphosphatidylinositol linkage; meanwhile, EphB receptors preferentially bind to ephrin-Bs (ephrinB1–ephrinB3), which are transmembrane proteins^1,2^. Among the Eph receptors, EphA4 is unique because it can interact with most ephrin-As and ephrin-Bs^3^.

EphA4 plays an essential role in different developmental processes and functioning—in particular, neuronal migration and neural circuit formation during brain development as well as synapse development and synaptic plasticity^4,5^. Deregulated expression or aberrant increased activity of EphA4 is reported in various human diseases such as Alzheimer’s disease (AD), amyotrophic lateral sclerosis, and cancers including breast cancer and pancreatic cancer, suggesting that EphA4 may be a promising drug target^6–9^. Therefore, identification of lead compounds as inhibitors that target EphA4 would be desirable for drug development^10^.

EphA4 comprises extracellular, transmembrane, and cytoplasmic regions. The extracellular region includes the ephrin ligand-binding domain (LBD), cysteine-rich domain, and fibronectin type III domain. Meanwhile, the cytoplasmic region contains the juxtamembrane region, tyrosine kinase domain, SAM domain, and PDZ target site^11^. Inhibitors of kinases can be designed on the basis of their ability to target the ATP pocket in the kinase domain at the active or inactive state or inhibiting the receptor–ligand interaction^10^. Given that the ATP-binding sites are well conserved among different Eph receptor members, it is challenging to identify inhibitors that are selective for EphA4.

Here, we identified small molecules that target the LBD of EphA4 for drug discovery. The whole extracellular domain of EphA4 is crystallized in its dimer or trimer form with or without ephrins^12^. This domain is composed of J-K and D-E loops that form complexes with its cognate ephrin ligands in a sandwich manner. While the D-E loop is always a beta-hairpin, the J-K loop adopts various conformations in different crystal structures. To date, there are three crystal structures of human EphA4 LBD available in the Protein Data Bank (PDB): one in apo form (PDB ID: 2WO1) and the other two in holo forms (PDB IDs: 2WO2 and 2WO3)^13^. These three structures of the EphA4 LBD are very similar, except for the J-K loop. The interaction of the LBD with ephrin naturally induces different conformations of the J-K loop, which is quite different from that in the apo form. Specifically, the J-K loop in 2WO1 is a beta-hairpin, the corresponding part in 2WO2 is a loop conformation with ephrin-B2, and that in 2WO3 is an alpha-helix secondary structure with ephrin-A2. Moreover, the distance between the J-K and D-E loops also varies, rendering different sizes of the binding sites.

Small molecule inhibitors of EphA4 with different scaffolds, e.g., 2,5-dimethylpyrrolyl benzene^14^ and rhynchophylline^6^, have been identified. Nonetheless, a major challenge for further drug development is the toxicity of lead compounds^15^. Repurposing of already-approved drugs for other indications may be an alternative for drug development^16^. This strategy is based on drug promiscuity/polypharmacology, which is the intrinsic nature of many compounds^17^. Several drugs have been successfully repurposed in past decades through the use of both *in silico* and *in vitro*/*vivo* methods^18–20^. Accordingly, in this study, we combined virtual screening and cellular assays to identify novel EphA4 inhibitors from among FDA-approved drugs.

## Results

We performed virtual screening for EphA4 inhibitor candidates using AutoDock Vina, a docking program that computationally examines the binding energy of a compound to its target. We ranked 1317 FDA-approved drugs according to their simulated binding energy. Based on a previously identified inhibitor of EphA4, rhynchophylline, whose docking energy is −9.0 kcal/mol^6^, we set the threshold to be −10.0 kcal/mol in order to obtain more potent candidates. As a result, we selected 43 compounds with a docking energy ≤−10.0 kcal/mol (Supplementary Table S1). Regarding their structures, most of these molecules had an elongated scaffold with several rings (Supplementary Fig. S1), indicating that they are complementary to the shape and hydrophobic nature of the binding site.

**Figure 1:**
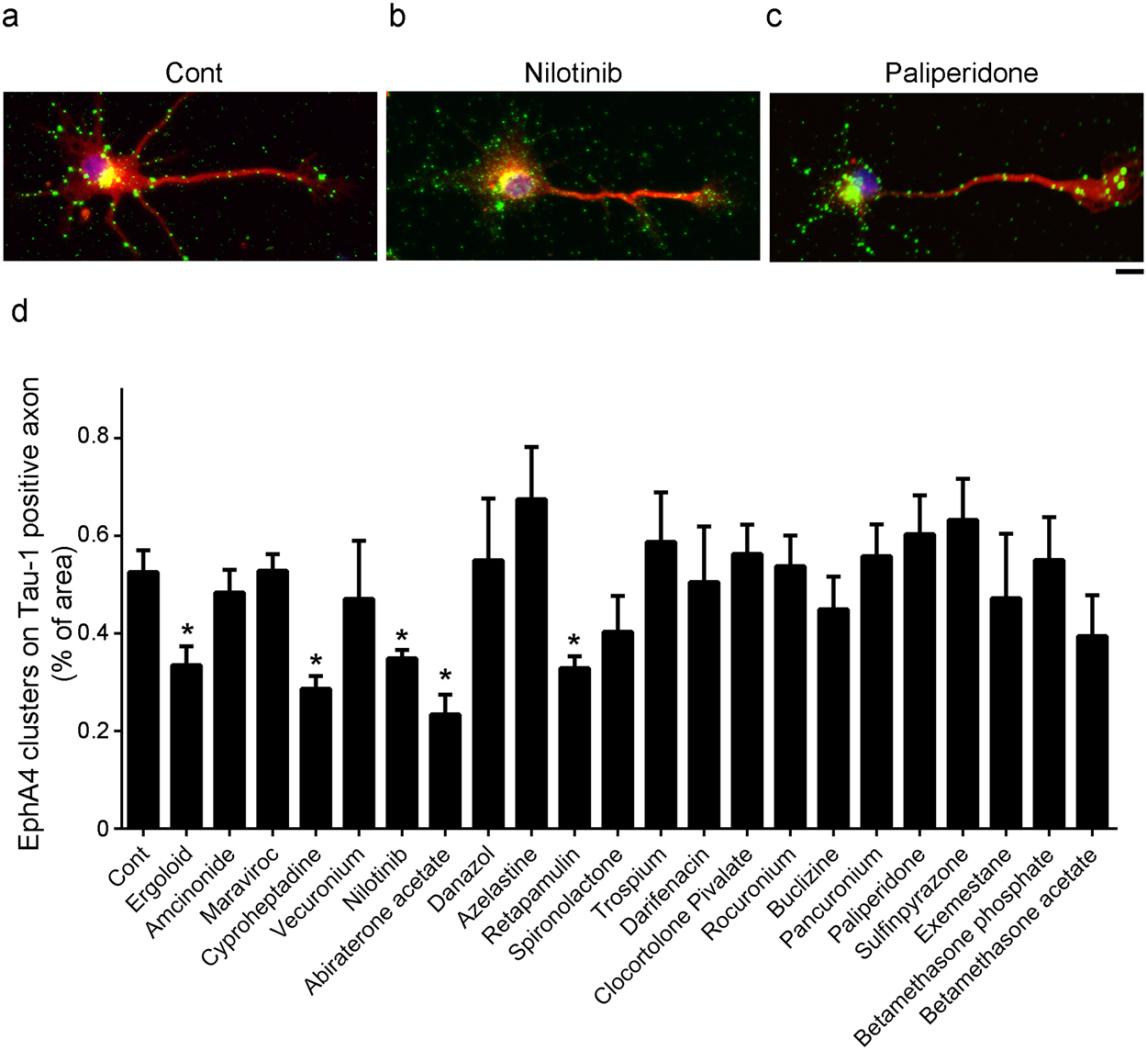
Inhibitory activity of 22 selected FDA-approved drugs in ephrin-A1–induced EphA4 clusters in cultured rat hippocampal neurons. Cultured hippocampal neurons at 3 days *in vitro* were treated with the candidate drugs (50 μM), followed by clustered ephrin-A1 (A1). The neurons were then subjected to immunostaining for EphA4 (green) and Tau-1 (red) antibodies. (**a**–**c**) Representative images of EphA4- and Tau-1–stained neurons. Ephrin-A1 (A1)-treated neurons without drug treatment (**a**), or with pretreatment with nilotinib (**b**) or paliperidone (**c**). (**d**) Quantitative analysis of EphA4 clusters on axons of ephrin-A1–treated neurons. Scale bar = 10 μm. *n* = 4 neuronal cell cultures; *p < 0.05 vs. Cont (ephrin-A1 treated neurons alone); unpaired Student’s *t*-test.

**Figure 2:**
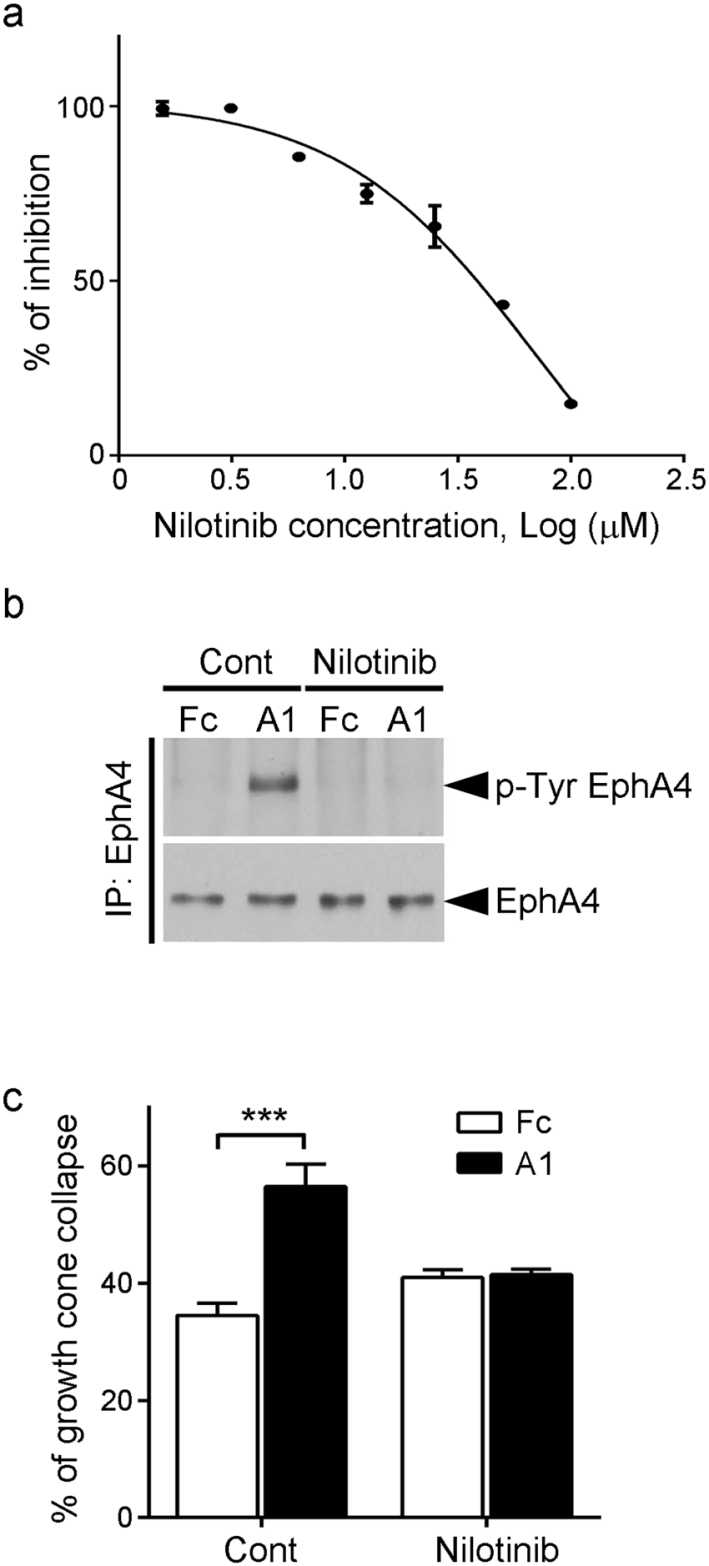
Nilotinib inhibits ephrin-A and EphA4 interaction, and EphA4-dependent signaling and cellular functions. (**a**) Dose–response curve of the inhibitory activity of nilotinib against the interaction of mouse EphA4 with ephrin-A1. (**b**) Nilotinib attenuated the ephrin-A1 (A1)-induced EphA4 tyrosine phosphorylation in rat hippocampal neurons. Lysate was immunoprecipitated with EphA4 antibody and subjected to western blot analysis for P-Tyr. (**c**) Nilotinib inhibited ephrin-A1–stimulated growth cone collapse in cultured hippocampal neurons (mean ± SEM, ≥75 neurons for each group from 3 experiments). ***p < 0.001; two-way ANOVA followed by the Bonferroni *post hoc* test. Cont (ephrin-A1–treated neurons alone).

We examined the inhibitory activities of 22 candidate EphA4 inhibitors using an ephrin-A1–induced EphA4 clustering assay in cultured hippocampal neurons. We found that five drugs—ergoloid, cyproheptadine, nilotinib, abiraterone, and retapamulin—inhibited the ephrin-A1–induced EphA4 clustering (by ~30% at a dose of 50 μM; Fig. 1). Specifically, nilotinib inhibited the interaction between the extracellular domain of mouse EphA4 and ephrin-A1 in a dose–dependent manner (IC_50_ = 70.67 µM; Fig. 2a). The effectiveness of nilotinib to inhibit EphA4 was further demonstrated in cellular context. Nilotinib essentially abolished ephrin-A1–induced tyrosine phosphorylation (Fig. 2b) and effectively blocked ephrin-A1–induced growth cone collapse (Fig. 2c) in hippocampal neurons.

**Figure 3:**
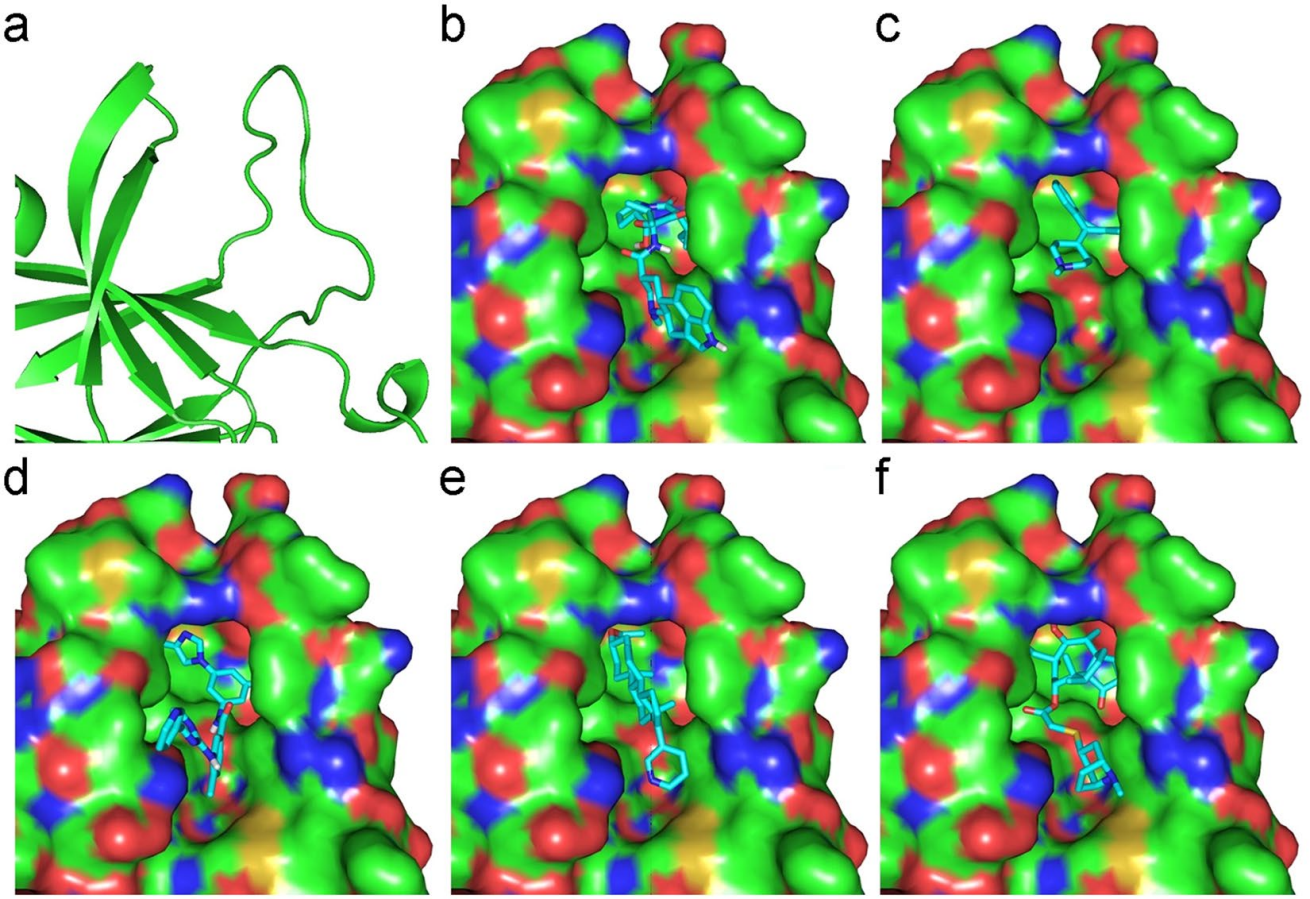
Molecular docking poses of five drugs in the cavity. Ligand-binding domain of EphA4 (**a**), and the docking conformations of ergoloid (**b**), cyproheptadine (**c**), nilotinib (**d**), abiraterone (**e**), and retapamulin (**f**) in the binding sites. The conformations with the lowest docking energy were selected for demonstration. The proteins are shown in surface representation (**b**–**f**), whereas drugs are shown as sticks.

The interactions of the EphA4 LBD with the five experimentally validated drugs are depicted in Fig. 3. Despite a few hydrogen bonds, binding was mainly achieved through hydrophobic interactions via several essential residues in the binding site of EphA4: F154, V157, and L166 in the J-K loop and I59 in the D-E loop. The binding of ergoloid to EphA4 is established by the hydrogen bond with D158 in the J-K loop and hydrophobic interactions with I59, F154, V157, and V195. Cyproheptadine binds EphA4 via pure hydrophobic interactions with I59, F154, V157, M164, L166, A193, and V195. Nilotinib is predicted to form hydrogen bonds with Q70 in the D-E loop and T104 as well as hydrophobic interactions with F154, V157, I163, L166, A193, and V195. The binding of abiraterone to EphA4 is achieved by a hydrogen bond with E62 in the D-E loop as well as hydrophobic interactions with I59, F154, V157, M164, L166, A193, and V195. Last, retapamulin forms a hydrogen bond with T104 and hydrophobic interactions with I59, F154, V157, L166, A193, and V195. To demonstrate the selectivity of these five experimentally validated drugs for EphA4, we conducted molecular docking analysis of these compounds towards other EphA and EphB receptors whose LBD crystal structures were available in the PDB. All five compounds bind EphA4 better than the other Eph receptors, with at least 1.1 kcal/mol less docking energy, suggesting that these compounds are selective EphA4 inhibitors (Supplementary Table S2).

## Discussion

In this study, we identified five FDA-approved compounds as EphA4 inhibitors. EphA4 is a promising target for different diseases, and blocking EphA4–ligand binding is a feasible therapeutic intervention approach for EphA4 inhibitors. Repurposing FDA-approved drugs is attractive because the safety and bioavailability of the agents have already been established.

The docking method has successfully identified rhynchophylline as an EphA4 inhibitor^6^, which exhibited EphA4 inhibitory activity in an *in vitro* assay and in an animal system. Importantly, rhynchophylline alleviates the synaptic dysfunction in an AD transgenic mouse model and other systems. The identification of five FDA-approved drugs as EphA4 inhibitors substantially contributes to drug discovery by increasing the number of scaffolds of potential inhibitors of EphA4.

Ergoloid is traditionally used to relieve the signs and symptoms of decreased mental capacity due to the aging process^21^. It is also used to treat some mood or behavior disorders, or other problems that may be due to changes in the brains of patients with AD or multiple small strokes^22^. Cyproheptadine is an H1-receptor antagonist and serotonergic antagonist^23^. Abiraterone acetate is combined with prednisone to treat patients with metastatic castration-resistant prostate cancer before chemotherapy^24^. Retapamulin is used for the treatment of bacterial skin infections such as impetigo^25^. As an antibacterial agent, specifically a protein synthesis inhibitor, retapamulin selectively inhibits bacterial protein synthesis by interacting at a site on the 50S subunit of the bacterial ribosome via an interaction that differs from those of other antibiotics^26^. Specifically, nilotinib, which demonstrated a promising IC_50_ in the ELISA experiment, is a kinase inhibitor that targets kinases including BCR-ABL, KIT, LCK, EPHA3, EPHA8, DDR1, DDR2, PDGFRB, MAPK11, and ZAK^27^.

Importantly, as these compounds are FDA-approved drugs, they have already passed many rigorous tests on safety, pharmacokinetics, and pharmacodynamics, which are essential in clinical trials. The biochemical and cellular assays validate that they are EphA4 inhibitors with potential to be further developed as repurposed drugs for new indications. Our integrated approach combining *in silico* and *in vitro/vivo* methods also demonstrated an efficient and successful application of drug repurposing for complicated diseases such as AD, thus shedding light on potential novel therapeutics.

## Conclusion

Our aim of this study is to identify new EphA4 inhibitors as prospective therapy for various neurodegenerative diseases. Thus, our combined approach for repurposing FDA-approved drugs was demonstrated to be promising, and the identified drug candidates offer new scaffolds for AD drug discovery.

## Methods

### Virtual screening of FDA-approved drugs

We first checked all of the FDA-approved small molecule drugs in DrugBank and then manually removed inorganic compounds, organic polymers, and those unsuitable for docking^28^. This step left us with 1317 drugs. To perform the virtual screening of these drugs with the EphA4 LBD, we utilized AutoDock Vina to estimate the binding energy of the ligand and target^29^. We selected a structure from 2WO2, an EphA4–ephrin-B2 complex with ephrin-B2 manually removed. The PDB IDs of the other Eph receptors were as follows: 3HEI for EphA2, 4L0P for EphA3, 4ET7 for EphA5, 3ETP for EphB2, and 2HLE for EphB4. AutoDock Tools was used to prepare the protein by merging nonpolar hydrogens and add Gasteiger partial charges^30^. We also prepared each drug with the assignments of partial charge and bond type. The docking grid box was centered between the J-K and D-E loops, with a volume of 22 × 20 × 22 Å. Finally, each docking simulation had 48 (parameter: exhaustive) parallel runs.

### EphA4 tyrosine phosphorylation, growth cone collapse, and EphA4 clustering assays

Primary hippocampal neurons were prepared from embryonic day 18–19 rat embryos as described previously^31^. We seeded rat hippocampal neurons on 48-well plates coated with poly-d-lysine (50 μg/mL; Sigma) at 6000 cells/well for the EphA4 clustering assay, 0.1 × 10^5^ cells/well (18 mm coverslip) for the growth cone collapse assay, and 5 × 10^6^ cells/60-mm plate for the EphA4 tyrosine phosphorylation assay. We incubated neurons with Neurobasal medium (Thermo Fisher Scientific) supplemented with 2% B27 (Thermo Fisher Scientific). We pretreated neurons at 3–4 days *in vitro* with the test compounds (50 μM) for 20 min, followed by pre-clustered ephrin-A1 Fc chimera (0.1–0.25 μg/mL; R&D Systems) or Fc (Jackson ImmunoResearch) as negative control of ephrin-A1 treated neurons for another 30 min.

For the EphA4 tyrosine phosphorylation assay, cultured hippocampal neurons were lysed in RIPA lysis buffer with various protease inhibitors. We then immunoprecipitated 500 μg protein lysates with EphA4 antibody (Santa Cruz Biotechnology) at 4 °C for 2 h, followed by incubation with protein G-Sepharose at 4 °C for 1 h. The samples were then washed with lysis buffer and resuspended in SDS sample buffer. Co-immunoprecipitated proteins were examined by western blot analysis as described previously^31^.

For the growth cone collapse assay, neurons were fixed with 4% paraformaldehyde and then incubated with Alexa Fluor 488-conjugated phalloidin (Molecular Probes) for filamentous actin labeling and anti–Tau-1 antibody (Millipore) for axon labeling^32^.

For the EphA4 clustering assay, the fixed neurons were immunostained with anti–EphA4 and anti–Tau-1 antibodies^6^. We performed the cellular imaging and quantitation of EphA4 clusters by using an IN Cell Analyzer 6000 high-content assay system (GE Healthcare). To segment the EphA4 clusters in axons, neurons were labeled with Hoechst and the axons were segmented according to Tau-1–positive signals. The EphA4 clusters in the axons were defined by the intensity segmentation method. The minimum threshold of the EphA4 signal in axons was determined in untreated neurons. To filter EphA4 clusters in the ephrin-A1–treated neurons, the maximum threshold was determined through direct sampling of the pixel intensities of the EphA4 clusters in the axons. An example of EphA4 cluster segmentation in ephrin-A1–treated neurons is shown in Supplementary Fig. S2. After the segmentation was set, all images obtained were analyzed at the same time to reduce variation among experiments. EphA4 clusters were quantified by calculating the proportion of the total area of EphA4 clusters in the axons (i.e., the Tau-1–positive area) of each well. Two independent experiments were performed. Each treatment was administered in duplicate for each experiment. Twenty fields with total of 50–100 neurons were obtained from each well of 48-well plate for automated analysis. To evaluate the algorithm performance of the automated IN Cell Investigator Image Analysis to quantify EphA4 clusters, nilotinib- or paliperidone-treated, or control conditions were selected for manual analysis. EphA4 clusters on the axons of neurons in the above conditions were quantified and using ImageJ as described previously (Supplementary Fig. S3)^6^. Ten to fifteen neurons from two experiments were randomly selected for quantitation. The EphA4 cluster results obtained from the automated and manual quantitative methods were comparable (Fig. 1b and Supplementary Fig. S3).

### ELISA

We immobilized recombinant mouse EphA4 ectodomain Fc chimera (R&D Systems) in buffer (25 mM sodium carbonate and 25 mM sodium bicarbonate, pH 9.7) on a Nunc MaxiSorp 96-well plate (eBioscience). We subsequently incubated the plate with biotinylated recombinant mouse ephrin-A1 in TBST (20 mM Tris-HCl [pH 7.6], 150 mM NaCl, 0.01% [v/v] Tween-20) at 30 °C for 1 h, followed by streptavidin-conjugated HRP (Thermo Scientific) for 1 h. We then incubated the plate with the substrate, TMB One Solution (Promega), until a blue color developed. We stopped the reaction by adding 1 N hydrogen chloride and measured the absorbance at 450 nm by using an ELISA plate reader^33^.

## Electronic supplementary material

Supplementary information
