# Supplementary material for: Identification of new EphA4 inhibitors by virtual screening of FDA-approved drugs
Source: Sci Rep. 2018 May 9;8:7377. doi: 10.1038/s41598-018-25790-1 (PMC5943255; doi:10.1038/s41598-018-25790-1)

SUPPLEMENTARY MATERIAL

Identification of new EphA4 inhibitors by virtual screening of FDA-approved drugs

Shuo Gu[1,2,3,†], Wing-Yu Fu[1,2,3,†], Amy K. Y. Fu[1,2,3,5], Estella Pui Sze Tong[1,2,3], Kwok-Wang Hung[1,2,3], Fanny C. F. Ip[1,2,3,5], Xuhui Huang[3,4], Nancy Y. Ip[1,2,3,5,*]

[1] Division of Life Science, [2] Molecular Neuroscience Center, [3] State Key Laboratory of Molecular Neuroscience, [4]Department of Chemistry, The Hong Kong University of Science and Technology, Clear Water Bay, Hong Kong, China

[5] Guangdong Provincial Key Laboratory of Brain Science, Disease and Drug Development, HKUST Shenzhen Research Institute, Shenzhen, China

[†] These authors contributed equally to this work.

[*] Correspondence and requests for materials should be addressed to N.Y.I. (email: boip@ust.hk)

Supplementary Figure S1 | 2-D structures of the 43 FDA-approved drugs selected from virtual screening

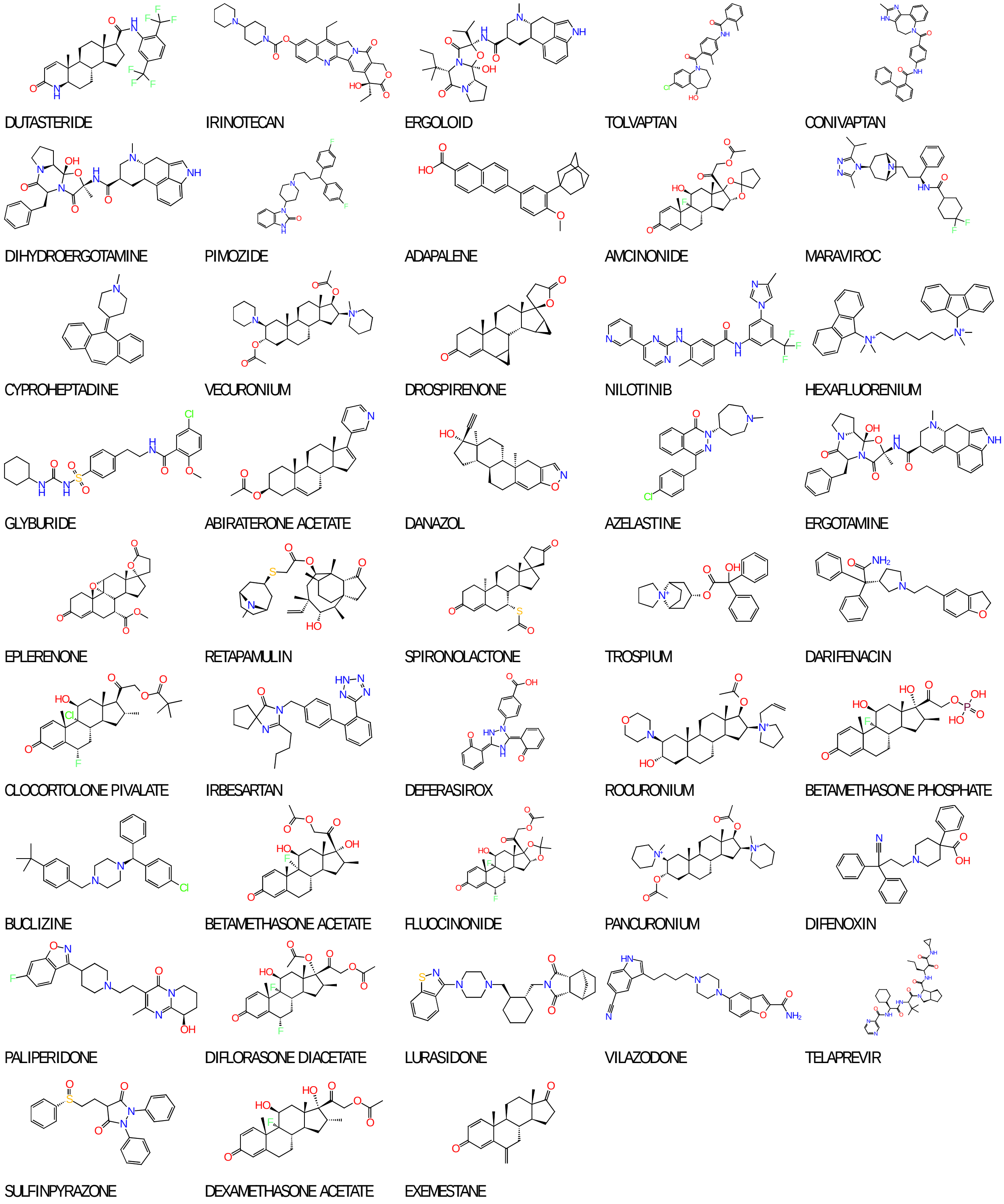

Supplementary Figure S2 | EphA4 clusters in ephrin-A1–treated hippocampal neurons. (a) Fluorescence image shows the EphA4 clusters (green), Tau-1–positive axon (red), and nucleus (blue) in ephrin-A1–treated hippocampal neurons. (b) Outline of the neuron to show the segmentation of the EphA4 clusters (circled by purple lines), axon identified by tau-positive signal (green line), and nucleus identified by DAPI (yellow line) after thresholding. White arrows: EphA4 clusters. Scale bar = 10 μm.

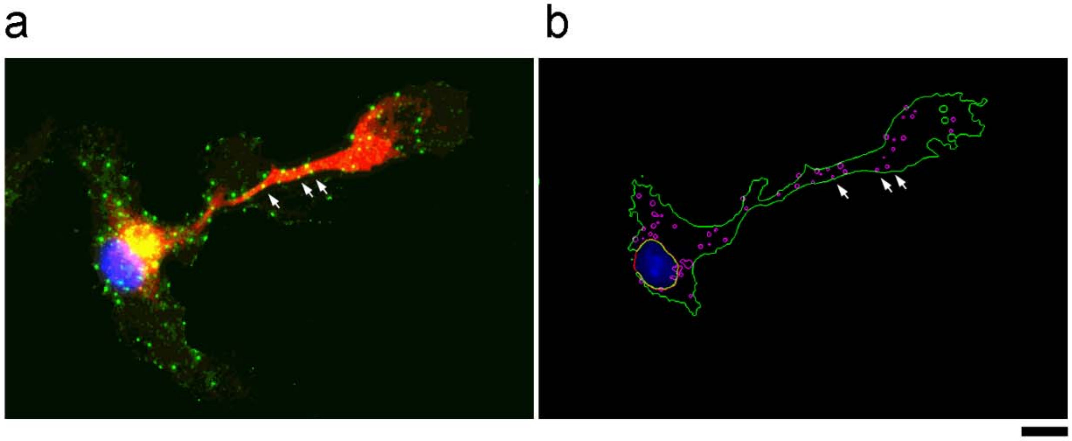

Supplementary Figure S3 | Nilotinib selectively inhibits ephrin-A1–induced EphA4 clusters. Quantification of EphA4 cluster density in axons of hippocampal neurons (3 days *in vitro*; mean ± SEM, 10–15 neurons for each group from 2 independent experiments). ***$p < 0.001$ vs. Control (neurons with ephrin-A1 treatment alone); unpaired Student's *t*-test.

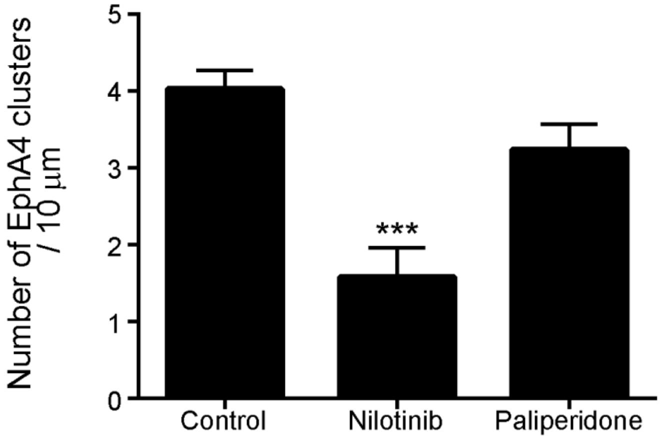

Supplementary Table S1 | Top 43 FDA-approved drugs ranked by their docking energy to the ligand-binding domain of EphA4

**Drug**	**Docking energy kcal/mol**
Dutasteride	−11.6
Irinotecan	−11.3
Ergoloid	−11.1
Tolvaptan	−11.0
Conivaptan	−10.8
Dihydroergotamine	−10.7
Pimozide	−10.7
Adapalene	−10.7
Amcinonide	−10.6
Maraviroc	−10.6
Cyproheptadine	−10.5
Vecuronium	−10.5
Drospirenone	−10.5
Nilotinib	−10.4
Hexafluorenium	−10.4
Glyburide	−10.4
Abiraterone acetate	−10.4
Danazol	−10.4
Azelastine	−10.4
Ergotamine	−10.4
Eplerenone	−10.4
Retapamulin	−10.4
Spironolactone	−10.3
Trospium	−10.3
Darifenacin	−10.2
Clocortolone pivalate	−10.2
Irbesartan	−10.2
Deferasirox	−10.2
Rocuronium	−10.1
Betamethasone phosphate	−10.1
Buclizine	−10.1
Betamethasone acetate	−10.1

Drug	Docking energy kcal/mol
Fluocinonide	−10.1
Pancuronium	−10.1
Difenoxin	−10.1
Paliperidone	−10.1
Diflorasone diacetate	−10.0
Lurasidone	−10.0
Vilazodone	−10.0
Telaprevir	−10.0
Sulfinpyrazone	−10.0
Dexamethasone acetate	−10.0
Exemestane	−10.0

Supplementary Table S2 | Docking energy (kcal/mol) of five experimentally validated ligands with available ligand-binding domains of Eph receptors.

**Drug**	**EphA4**	**EphA2**	**EphA3**	**EphA5**	**EphB2**	**EphB4**
Ergoloid	−11.1	−8.6	−9.4	−8.3	−5.7	−7.3
Cyproheptadine	−10.5	−8.3	−8.7	−7.4	−5.9	−6.8
Nilotinib	−10.4	−8.3	−9.3	−8.3	−7.7	−8.1
Abiraterone	−10.4	−8.5	−8.7	−7.2	−5.0	−7.7
Retapamulin	−10.4	−7.7	−8.6	−7.7	−6.2	−7.5

Unprocessed original scans of western blots shown in Figure 2b. Nilotinib attenuated the ephrin-A1 (A1)-induced EphA4 tyrosine phosphorylation in rat hippocampal neurons.

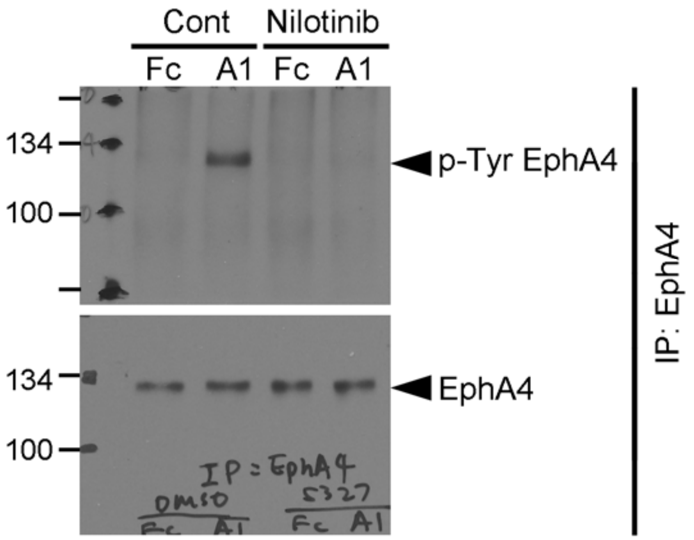

Supplement: Supplementary file 1 — Supplementary information [file 41598_2018_25790_MOESM1_ESM.pdf]
